# Prevailing I292V PB2 mutation in avian influenza H9N2 virus increases viral polymerase function and attenuates IFN-β induction in human cells

**DOI:** 10.1099/jgv.0.001294

**Published:** 2019-07-15

**Authors:** Weihua Gao, Zhipeng Zu, Jiyu Liu, Jingwei Song, Xinyu Wang, Chenxi Wang, Litao Liu, Qi Tong, Mingyang Wang, Honglei Sun, Yipeng Sun, Jinhua Liu, Kin-Chow Chang, Juan Pu

**Affiliations:** ^1^ Key Laboratory of Animal Epidemiology, Ministry of Agriculture, College of Veterinary Medicine, China Agricultural University, Beijing, 100193, PR China; ^2^ School of Veterinary Medicine and Science, University of Nottingham, Sutton Bonington Campus, Loughborough, UK

**Keywords:** PB2 mutation, H9N2 influenza virus, Mammalian infectivity, Polymerase activity, Beta interferon expression

## Abstract

Adaptation of PB2 protein is important for the establishment of avian influenza viruses in mammalian hosts. Here, we identify I292V as the prevalent mutation in PB2 of circulating avian H9N2 and pandemic H1N1 viruses. The same dominant PB2 mutation is also found in most human isolates of emergent avian H7N9 and H10N8 viruses. In human cells, PB2-292V in H9N2 virus has the combined ability of conferring higher viral polymerase activity and stronger attenuation of IFN-β induction than that of its predecessor PB2-292I. IFN-β attenuation is accompanied by higher binding affinity of PB2-292V for host mitochondrial antiviral signalling protein, an important intermediary protein in the induction of IFN-β. In the mouse *in vivo* model, PB2-292V mutation increases H9N2 virus replication with ensuing increase in disease severity. Collectively, PB2-292V is a new mammalian adaptive marker that promotes H9N2 virus replication in mammalian hosts with the potential to improve transmission from birds to humans.

## Introduction

Avian H9N2 influenza viruses circulate worldwide and are endemic in terrestrial avian species [1, 2]. Sporadically, they are transmitted to mammalian species, including humans and pigs [3, 4]. Recently, Li *et al.* found that the H9N2 viruses isolated between 2009 and 2013 showed enhanced virulence to ferrets and have acquired efficient respiratory droplet transmission between ferrets without prior adaptation [5]. Based on the World Health Organization data, human cases of H9N2 virus infection have also increased in recent years. In the previous six years (2013–2018), 33 human cases were laboratory-confirmed in China, making up 76 % (33/43) of the national H9N2 cases (http://www.who.int/influenza/human_animal_interface/HAI_Risk_Assessment/en/). Since 2013, two novel reassortants, H7N9 and H10N8 viruses, whose internal genes were derived from prevailing H9N2 virus, have caused regular human infections and prompted serious public health concerns [6–8]. Together, current avian H9N2 viruses and their reassortants exhibit increasing transmissibility in humans, indicating that their shared H9N2-derived internal genes are progressively adapting in human replication.

PB2 protein, one of the subunits of the ribonucleoprotein (RNP) complex, is a major virulence and host range determinant of influenza viruses [9]. The avian origin PB2 gene in influenza viruses generally exhibits restricted polymerase activity and impaired virus growth in mammalian hosts [9–13]. To overcome this restriction, the avian PB2 gene needs to acquire certain mutational changes to improve its activity in mammalian cells, as exemplified in PB2-E627K, -D701N, -T271A, -K526R and -A588V [10, 14–18]. Additionally, it was recently reported that PB2 also participates in regulating the host innate immune responses through inhibiting IFN-β expression [19]. Collectively, these findings suggest that the PB2 gene has developed multiple strategies to adapt to mammalian hosts.

Here, we identified I292V mutation in PB2 as a dominant genotype in avian H9N2 (since 2010), and in emergent H7N9 and H10N8 viruses; human isolates of such viruses frequently contain the I292V PB2 genotype. Furthermore, the amino acid residue PB2-292V is predominant in pandemic (pdm) H1N1 human viruses. The PB2-I292V in H9N2 virus confers increased replication and more severe pathogenicity to mice through enhancing viral polymerase and strong attenuation of IFN-β induction. Therefore, PB2-I292V is a newly identified mammalian adaptive determinant of avian H9N2 influenza viruses.

## Methods

### Viruses, plasmids and cells

A/chicken/Shandong/lx1023/2007 (Lx1023) (H9N2) virus was isolated from diseased chickens in Shandong, China, in October 2007. The virus was propagated in 9- to 11-day-old specific pathogen-free (SPF) embryonated chicken eggs. pcDNA-PB2, pcDNA-PB1, pcDNA-PA and pcDNA-NP expression plasmids of H9N2 were generated by subcloning the corresponding coding segment into pcDNA3.1 vector. pRK5-Flag-MAVS was generated by subcloning the human mitochondrial antiviral signalling (MAVS) cDNA into pRK5-Flag.

Human embryonic kidney (293T), Madin–Darby canine kidney (MDCK), human lung adenocarcinoma epithelial (A549) and chicken fibroblast (DF-1) cells were cultivated in Dulbecco’s modified Eagle’s medium (DMEM, Invitrogen) supplemented with 10 % fetal bovine serum, 100 units ml^−1^ of penicillin and 100 µg ml^−1^ of streptomycin. All cell cultures were kept at 37 °C and 5 % CO2.

### Polymerase activity assay

A dual-luciferase reporter assay system (Promega, Madison, WI, USA) was used to compare the polymerase activities of viral RNP complexes. The PB2, PB1, PA and NP gene segments of wild-type viruses and the mutated PB2 gene were cloned into the pcDNA3.1 expression plasmid. The PB2, PB1, PA and NP plasmids (125 ng each plasmid) were used to transfect 293 T or DF1 cells, together with a pYH-NS1-Luci plasmid expressing a reporter firefly luciferase gene under the control of the human or chicken RNA polymerase I promoter (10 ng), and an internal control plasmid expressing Renilla luciferase (2.5 ng). Cultures were incubated at 33, 37 or 39 °C. Cell lysates were analysed 24 h after transfection to measure firefly and Renilla luciferase activities using GloMax 96 microplate luminometer (Promega).

### IFN-β assay

The 293T cells in 24-well plates were transfected with 200 ng of IFN-β firefly luciferase plasmid, 20 ng Renilla luciferase internal control plasmid, and 400 ng of the influenza virus PB2-expressing plasmid, GFP-expressing plasmid or pcDNA3.1 vector. At 18 h post transfection, 293T cells were inoculated with Sendai virus to activate the IFN-β signalling. The cells were incubated for 18 h and then lysed in 100 µl of passive lysis buffer (PLB; Promega). Firefly and Renilla luciferase activities were assessed using a Dual-Luciferase Assay Kit (Promega).

To determine the effect of PB2 protein on the expression of IFN-β mRNA, 293T cells in 24-well plates were transfected with 400 ng of the influenza virus PB2-expression plasmid, GFP-expressing plasmid or pcDNA3.1 vector. At 18 h post transfection, 293T cells were infected with Sendai virus to activate the IFN-β signalling. Total RNA was extracted from transfected 293T cells at 18 h post inoculation (h p.i.). To measure the levels of IFN-β expression during inﬂuenza virus infection, A549 cells were infected with H9N2 viruses at an m.o.i. of 2. Total cellular RNA was isolated using the TRIzol reagent (Invitrogen) at 12 and 24 h p.i. Total RNA from transfected 293T cells or influenza virus infected A549 cells was reverse transcribed using SuperScript II (Invitrogen) and oligo dT primer. The qRT-PCR mixture for each reaction sample consisted of 10 µl of 2×SYBR green PCR master mix (Applied Biosystems), 7 µl of nuclease-free water, 0.5 µl of each primer and 2 µl of cDNA template. Expression of IFN-β was quantified using the 7500 real-time PCR system (Applied Biosystems) and data was analysed using the 2^–△△*CT*^ method. Each experiment comprised three technical replicates for each sample, and three experimental replicates were performed.

### Western blotting

Total cell protein lysates were extracted from 293T cells with RIPA lysis buffer and total protein concentration was determined by a BCA protein assay kit (Beyotime, China). Protein samples derived from cell lysates were heated at 100 °C for 10 min and then separated on a 10 % sodium dodecyl sulfate-polyacrylamide (SDS-PAGE) gel and transferred to a polyvinylidene difluoride (PVDF) membrane (Bio-Rad, USA), and subsequently incubated with the appropriate primary antibody. Primary antibodies used were for the detection of β-actin (Beyotime, China), Flag (Sigma, USA), influenza virus PB2 (ThermoFisher, USA) and GFP (Santa Cruz, USA) protein. The secondary antibody used was horseradish peroxidase (HRP)-conjugated anti-rabbit or -mouse antibody (Beyotime, China). HRP presence was detected using a Western Lightning chemiluminescence kit (Amersham Pharmacia, Freiburg, Germany), following the manufacturer’s protocols.

### Immunoprecipitation assay

The 293T cells were transfected with MAVS and PB2 expression plasmids and incubated at 37 °C. At 24 h post-transfection, cells were collected and washed in ice-cold PBS. Immunoprecipitation of PB2 protein were performed as described previously [20]. In brief, cells were exposed to lysis buffer (50 mM Tris-Cl at pH 8.0, 150 mM NaCl, 1 % Triton X-100, 1 mM DTT, 1 complete protease inhibitor cocktail and 10 % glycerol). The clarified cell lysate was used for immunoprecipitation for 4 h with ANTI-FLAG M2 magnetic beads (Sigma). Beads were washed and boiled. Protein samples were analysed by Western blotting.

### Generation of recombinant viruses

All eight gene segments amplified by RT-PCR from H9N2 viruses were cloned into a dual-promoter plasmid, pHW2000. Mutation in the PB2 gene was introduced using Site-directed Quick Change Mutagenesis kit (Agilent) according to the manufacturer's instructions. Primer sequences are available upon request. All of the constructs were sequenced to confirm the site-directed mutation. Viruses were rescued using the eight-plasmid rescue system [21]. Briefly, all eight plasmids encoding gene segments were transfected in 293T cells using Lipofectamine 2000 (Invitrogen) according to the manufacturer’s instructions. After incubation for 6 h at 37 °C, the transfection mixture was removed from the cells, and 1 ml of Opti-MEM containing 2 µg ml^−1^ tosylsulfonyl phenylalanyl chloromethyl ketone-treated trypsin (TPCK-treated trypsin) was added. After 48 h, the harvested cell supernatant was used to inoculate 9-day-old SPF chicken embryos to produce stock viruses.

### Quantitative real-time PCR (qRT-PCR)

Levels of mRNA and vRNA were determined in A549 cells infected with H9N2 viruses. Total RNA was extracted from infected A549 cells using TRIzol reagent according to the manufacturer’s instructions (Invitrogen). For the detection of mRNA and vRNA, oligo dT primer and uni-12 primer (5**′**-AGCAAACGACC-3**′**) were respectively used to generate cDNAs from 1 µg of total RNA per sample using Superscript III First-Strand Synthesis SuperMix (Invitrogen). The qRT-PCR mixture for each reaction sample consisted of 10 µl of 2×SYBR green PCR master mix (Applied Biosystems), 7 µl of nuclease-free water, 0.5 µl of each primer and 2 µl of cDNA template. Expression of mRNA and vRNA of the β-actin, matrix protein (M1) and nucleoprotein (NP) genes was detected using the 7500 real-time PCR system (Applied Biosystems) with the following program: 1 cycle at 95 °C for 10 min, followed by 40 cycles of 95 °C for 15 s and 60 °C for 1 min. Expression values of each gene, relative to β-actin, were calculated using the 2^–△△*CT*^ method. Each experiment comprised three technical replicates for each sample, and three experimental replicates were performed. Primers for amplification of β-actin, M1 and NP genes are listed as follows: β-actin, forward, 5**′**-AGAGCTACGAGCTGCCTGAC-3**′**, reverse, 5**′**-CGTGGATGCCACAGGACT-3**′**; M1, forward, 5**′**-CGCACAGAGACTTGAGGATG-3**′**, reverse, 5**′**-TGGGTCTCCATTCCCATTTA-3**′**; NP, forward, 5**′**-CAGTGAAGGGGATAGGGACA-3**′**, reverse, 5**′**-CCAGGATTTCTGCTCTCTCG-3**′**.

### Virus titration and replication kinetics

TCID_50_ was determined in MDCK cells with tenfold serially diluted viruses inoculated at 37 °C for 48 h. The TCID_50_ value was calculated by the Reed–Muench method [22]. Multistep replication kinetics were determined by inoculating A549 or MDCK cells. After 1 h incubation at 37 °C, the cells were washed twice and further incubated in serum-free DMEM containing 2.0 µg ml^−1^ TPCK-treated trypsin. Cell supernatants were harvested at 12, 24, 36, 48, 60 and 72 h p.i. and titrated by inoculating MDCK cells. Each experiment was performed in triplicate.

### Mouse experiments

Groups of 6-week-old female BALB/c mice (Beijing Experimental Animal Center) were anesthetized with Zoletil 50 (tiletamine-zolazepam; Virbac S.A., Carros, France; 20 µg g^−1^) and inoculated intranasally with 50 µl PBS or 10^6^ TCID_50_ of H9N2 viruses in 50 µl PBS. Five mice from each group were monitored daily for 14 days, and mice losing more than 25 % of their original body weight were humanely euthanized. Three mice from each group were euthanized on 3, 5 and 7 days p.i. for virus titration and histopathology assay. Lungs were collected and homogenized in 1 ml of cold PBS. Virus titrer were determined by TCID_50_. A portion of the lung from each euthanized mouse at 5 days p.i. was fixed in 10 % phosphate-buffered formalin for histopathological examination, which was performed as described previously [23].

### Statistical analyses

All statistical analyses were performed using GraphPad Prism software version 5.00 (GraphPad Software, San Diego, CA, USA). Statistically significant differences between experimental groups were determined using ANOVA. Differences were considered statistically significant at *P*<0.05.

## Results

### Potential mammalian adaptative mutation of PB2-I292V is predominant in avian H9N2 influenza viruses

**Fig. 1.**
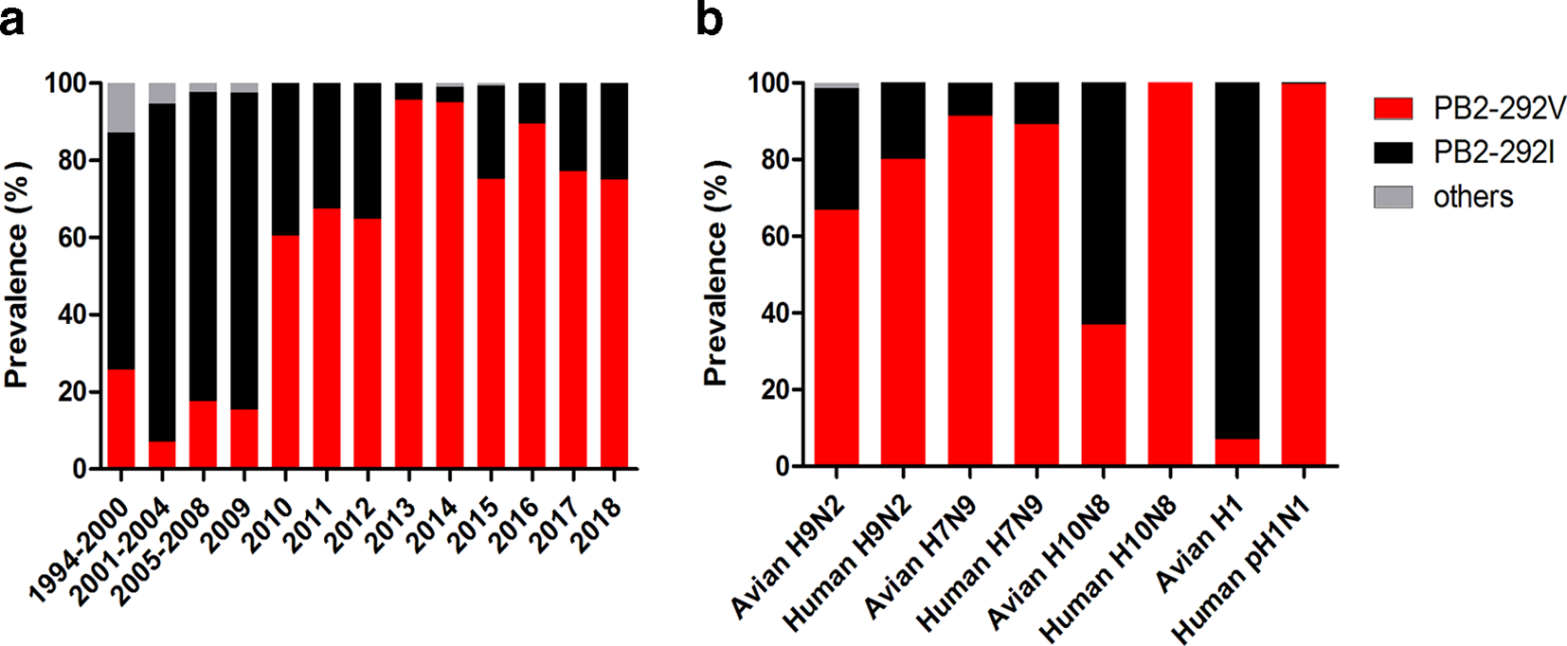
Prevalence of PB2-I292V in multiple subtypes of influenza viruses. (a) Prevalence of PB2-292V in avian H9N2 influenza viruses during the period of 1994–2018. (b) Prevalence of PB2-292V in avian and human H9N2, H7N9, H10N8, avian H1 and human pH1N1 influenza viruses during the period of 1994–2018. For each column from left to right, the actual number of viruses is 62, 56, 125, 39, 38, 83, 125, 329, 99, 165, 19, 35 and 4, respectively in (a); 1177, 15, 700, 1287, 46, 5, 85 and 710 in (b). Others refer to viruses without PB2-292I or -292V. The proportion of viruses with others (0.14 %) is too low to be seen in the third column from left to right (b).

Current avian H9N2 influenza virus and its reassortants exhibit a growing trend toward enhanced human infection capacity; adaptive mutations of PB2 protein have been demonstrated to be important in host adaptation [10, 16–18, 24, 25]. In this study, sequence analysis of PB2 proteins of H9N2 avian influenza viruses (AIVs) was performed to identify potential mammalian adaptive amino acids. The results showed that the H9N2 PB2-292 amino acid residue had undergone a shift from I to V over the past 20 years in China (Fig. 1a). During the period of 1994–2009, H9N2 viruses with PB2-292I were prevalent while viruses with PB2-292V were infrequently found. However, the frequency of H9N2 viruses with PB2-292V increased sharply from 15.38 % prevalence in 2009 to 60.53 % in 2010 to become predominant among avian H9N2 viruses. By the end of 2013, viruses possessing PB2-292V have become the absolute dominant circulating H9N2 (97.69 %) in avian throughout China (Fig. 1a). We further compared the prevalence of PB2-292V in H9N2, novel H7N9 and H10N8 viruses isolated from avian and human hosts (Fig. 1b). PB2-292V was dominantly prevalent in H7N9 viruses isolated from avian and human hosts, while H9N2 and H10N8 isolated from humans showed higher frequency of PB2-292V than from corresponding avian isolates. In addition, we found that PB2-292V was highly conserved in human pandemic H1N1 influenza viruses, while H1 influenza subtype isolated from avian species preferred to encode PB2-292I. These results indicate that PB2-292V is a potential mammalian adaptive amino acid residue, which might facilitate virus replication and pathogenicity in humans.

### PB2-I292V confers in H9N2 virus higher polymerase activity in human cells

**Fig. 2.**
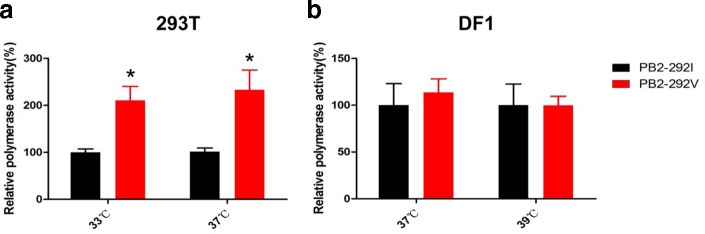
RNP polymerase activity was higher in the presence of PB2-292V than PB2-292I in human 293T cells. RNP polymerase activities of wild-type and mutant viruses in (a) 293T and (b) avian DF1 cells, transfected with polymerase plasmids (PB1, PA, NP and PB2-292I or PB2-292V), were determined by mini genome replication assays at different temperatures with reference PB2-292I at 100 %. Data are presented as means±sd of three independent experiments. Statistical significance was based on two-way ANOVA (*P<0.05, **P<0.01, ***P<0.001).

PB2 protein is a component of the ribonucleoprotein complex, which is responsible for viral polymerase activity and determines viral infection in new hosts [9, 26]. To identify whether PB2-I292V enhances the RNP polymerase activity, viral mini-genome polymerase assays were performed by transfecting wild-type PB2 (PB2-292I) or mutant PB2 (PB2-292V) gene encoding plasmid with PB1, PA and NP expression plasmids of wild-type H9N2 virus. As shown in Fig. 2a, PB2-I292V mutation resulted in significant increase in polymerase activity in human 293T cells at both 33 and 37 °C (*P*<0.05). However, in avian DF-1 cells, RNP polymerase activities with wild-type and 292V site-mutant PB2 showed no significant difference at 37 and 39 °C (Fig. 2b). These results demonstrate that PB2-292V is a host species-specific adaptor, which may specifically enhance the polymerase activity of H9N2 avian inﬂuenza virus in human cells.

### PB2-I292V augments transcription and genomic RNA replication of H9N2 virus in human cells

**Fig. 3.**
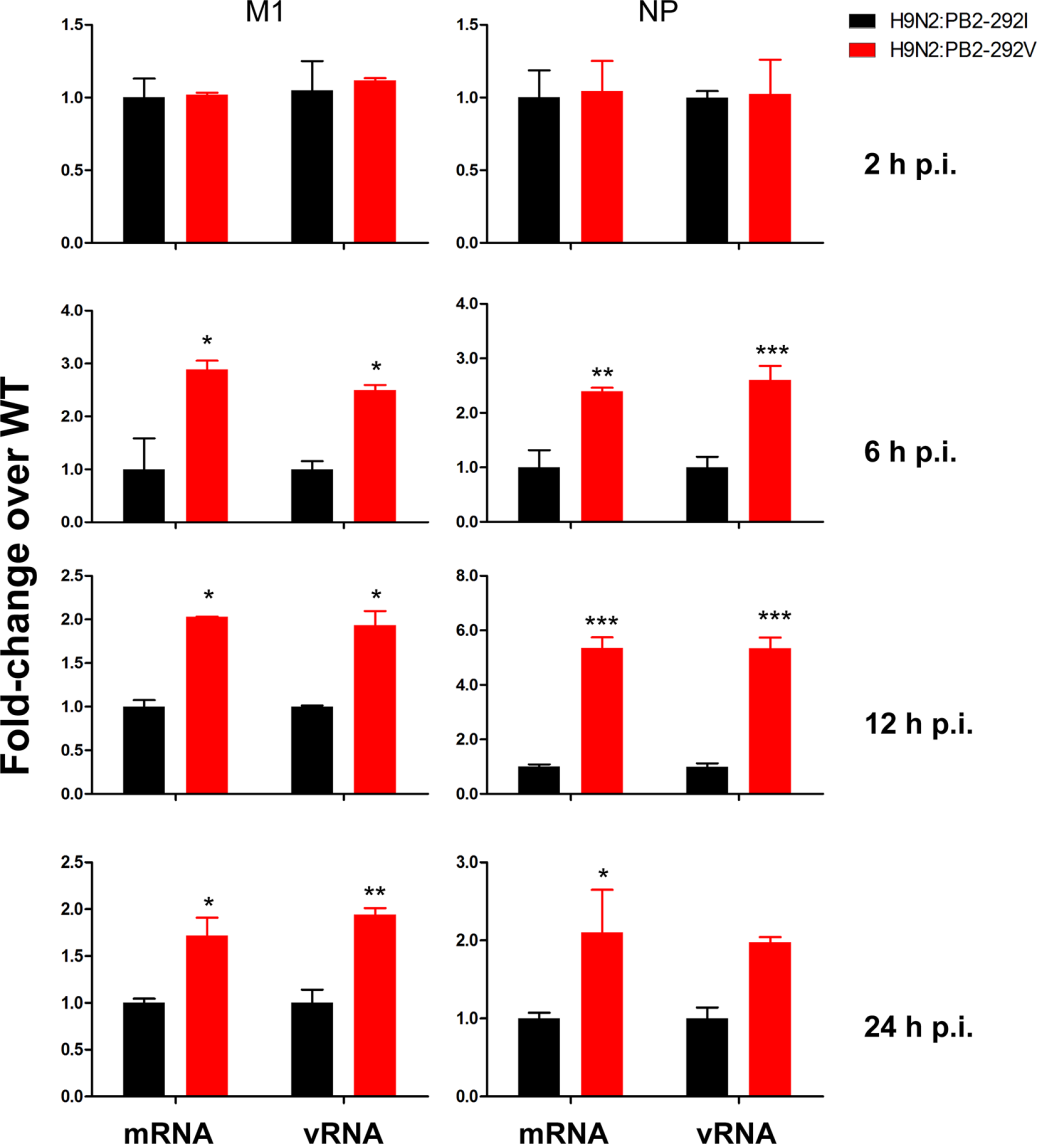
Avian H9N2 virus with PB2-292V generated more mRNA and vRNA of viral M1 and NP genes than the same virus with PB2-292I. A549 cells were infected separately with H9N2:PB2-292I and H9N2:PB2-292V at 0.2 m.o.i. for 2, 6, 12 and 24 h. Normalized mRNA and vRNA levels of M1 and NP at each time point are expressed as fold-change relative to H9N2:PB2-292I. Data are presented as means±sd of three independent experiments. Statistical significance was based on two-way ANOVA (*P<0.05, **P<0.01, ***P<0.001).

vRNP polymerase activity catalyses viral genomic transcription and replication [9]. To ascertain whether vRNP polymerase activity is affected by PB2-I292V mutation, we produced wild-type (H9N2:PB2-292I), and mutant (H9N2:PB2-292V) viruses by reverse genetics. Levels of viral mRNA and vRNA in A549 cells infected with wild-type and mutant viruses were determined at 2, 6, 12, 24 h p.i. (Fig. 3). The results of qRT-PCR showed that H9N2:PB2-292V produced significantly higher levels of both mRNA and vRNA of viral M1 and NP than H9N2:PB2-292I at 6, 12 and 24 h p.i. (*P*<0.05), demonstrating that PB2-I292V increases avian H9N2 influenza viral transcription and replication in human A549 cells.

### PB2-I292V mutational change in PB2 strongly attenuates virus induced IFN-β expression in human cells

**Fig. 4.**
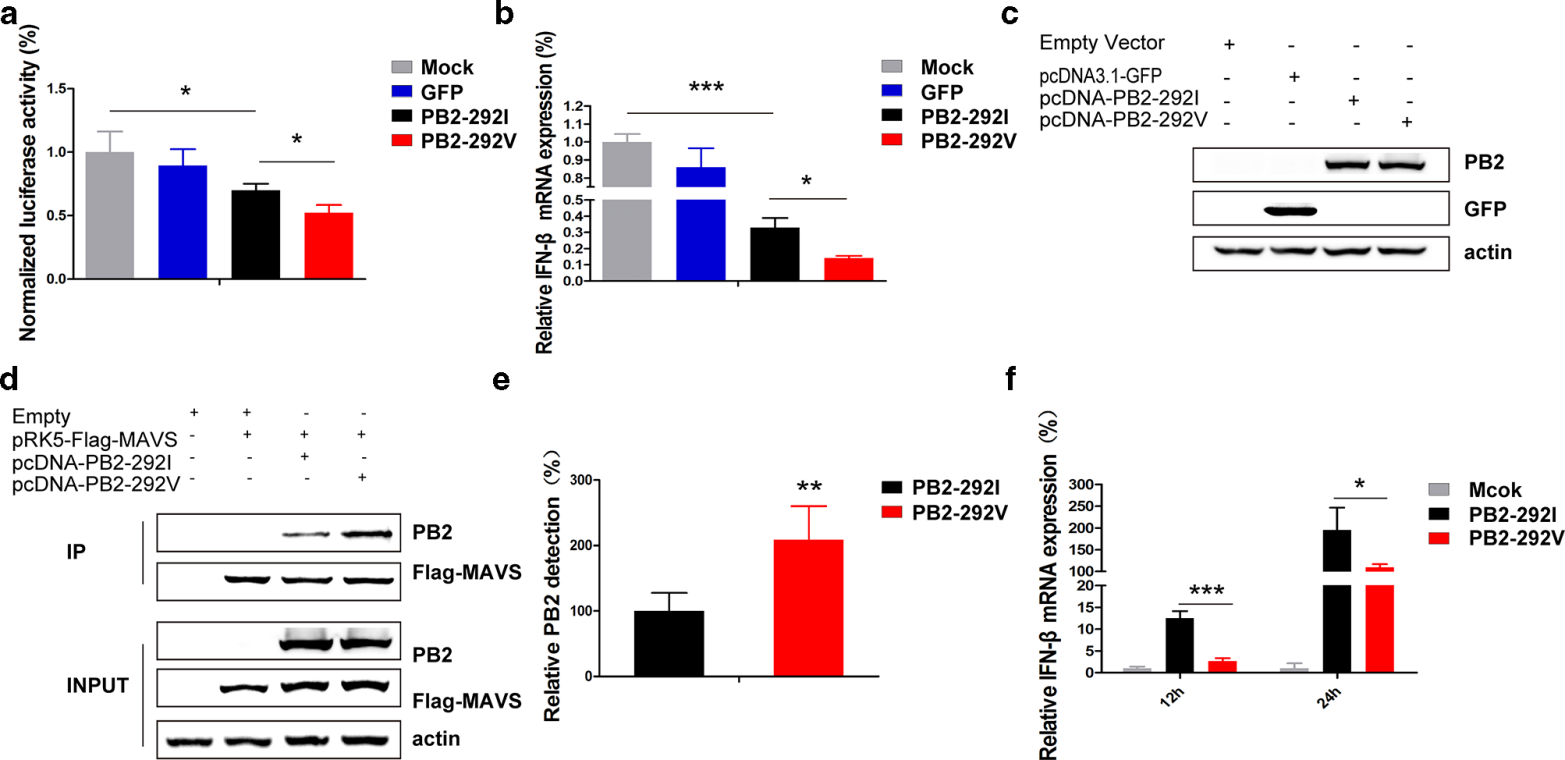
PB2-I292V mutation in avian H9N2 virus strongly attenuated IFN-β induction. (a) 293T cells were co-transfected with a human IFN-β promoter-luciferase reporter construct. (a) Renilla luciferase control plasmid, and indicated PB2 expression constructs or control GFP-expressing plasmid. At 18 h post-transfection, 293T cells were infected with Sendai virus and dual luciferase assay was performed at 18 h p.i. (b) Corresponding IFN-β expression levels normalized to β-actin were determined by qPCR. (c) Protein detection of PB2-292I and PB2-292V in 293T cells transfected with corresponding expression constructs. β-actin detection indicated uniform protein loading. (d) 293T cells were co-transfected with pRK5-Flag-MAVS, PB2-292I, PB2-292V and control constructs as indicated. Cells were lysed 24 h post-transfection and immunoprecipitation was performed with the anti-Flag M2 magnetic beads which showed co-immunoprecipitation of PB2 proteins. (e) Densitometry measurements using Image J showed higher co-immunoprecipitation of PB2-292V than PB2-292I. Relative reference value of PB2-292I protein was set at 100 %. Data are presented as means±sd of three independent co-immunoprecipitation experiments. (f) A549 cells were infected with H9N2:PB2-292I or H9N2:PB2-292V virus at an m.o.i. of 2 for 12 and 24 h. Stronger attenuation of IFN-β expression, as determined by qPCR normalized to β-actin, was evident with PB2-292V compared with PB2-292I virus. Data are presented as mean±sd of three independent experiments Statistical significance was based on two-way ANOVA (*P<0.05, **P<0.01, ***P<0.001).

PB2 protein was recently demonstrated to interfere with host IFN-β antiviral response [19, 27]. We compared the promoter response of human IFN-β to Sendai virus infection in the presence of PB2-292I and PB2-292V proteins in 293T cells (Fig. 4a). Both PB2-292I and PB2-292V significantly reduced IFN-β induction with PB2-292V conferring stronger attenuation (*P*<0.05) (Fig. 4a). In addition, corresponding IFN-β mRNA levels were reduced; in the presence of PB2-292V, IFN-β expression was 43 % of that of PB2-292I (*P*<0.05) (Fig. 4b). The observed increase in IFN-β inhibition with PB2-292V was not due to a difference in PB2 protein production as determined by Western blotting (Fig. 4c).

Previous study demonstrated that PB2 protein inhibited the expression of IFN-β by interacting with MAVS protein [19]. We next examined the relative binding of PB2-292V and PB2-292I to MAVS by co-transfection followed by immunoprecipitation (Fig. 4d, e). Both PB2-292I and PB2-292V co-immunoprecipitated with Flag-MAVS fusion protein; PB2-292V was co-immunoprecipitated at twice the level of PB2-292I, demonstrating that PB2-I292V mutation increases the binding affinity of PB2 to MAVS.

To further investigate whether the PB2-I292V mutation has an effect on the expression of IFN-β mRNA in response to influenza virus infection, A549 cells were separately infected with H9N2:PB2-292I and H9N2:PB2-292V virus and the IFN-β mRNA levels were determined at 12 and 24 h p.i. (Fig. 4f). IFN-β expression in H9N2:PB2-292V virus infected cells was significantly lower than those infected with H9N2:PB2-292I virus-infected cells (*P*<0.05). In summary, PB2-292V shows higher binding affinity for MAVS and is better at attenuating the induction of IFN-β than PB2-292I in H9N2 virus.

### PB2-I292V mutation increases replication of H9N2 virus in mammalian cells

**Fig. 5.**
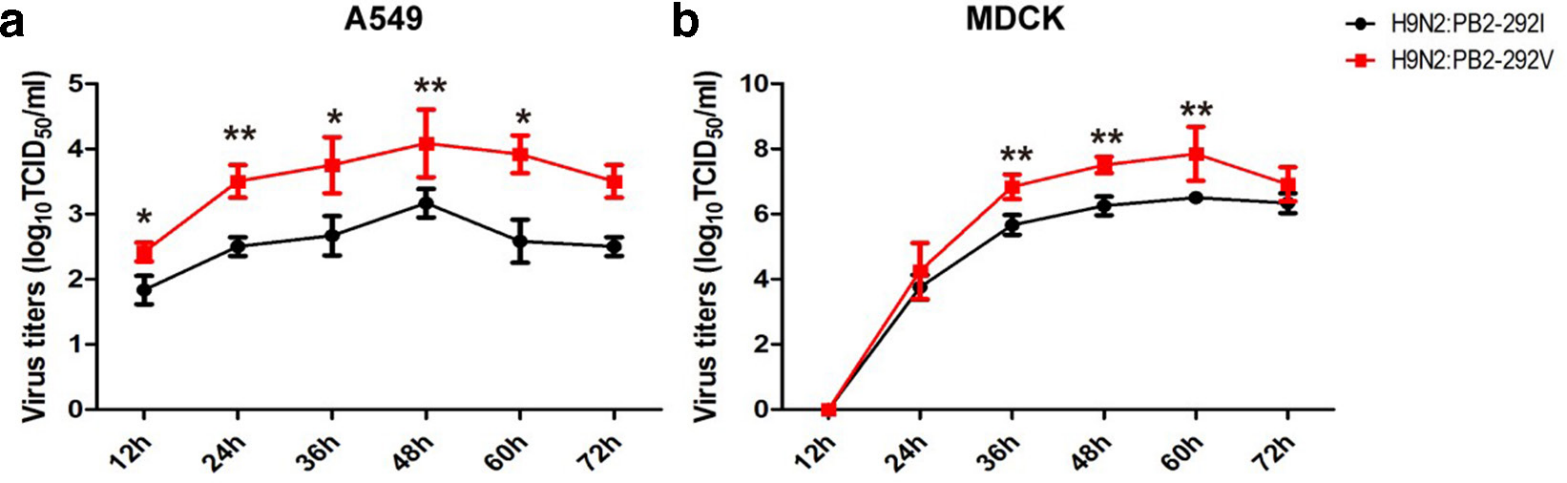
Avian H9N2 virus with PB2-292V replicated more effectively than the same virus with PB2-292I in (a) A549 and (b) MDCK cells. Multistep growth curves of H9N2 viruses were determined in A549 and MDCK cells at an m.o.i. of 0.2 and 0.001, respectively. Virus titres were determined from supernatants collected at the indicated time points. Data are presented as means±sd of three independent experiments. Statistical significance was based on two-way ANOVA (*P<0.05, **P<0.01, ***P<0.001).

Multicycle growth kinetics of wild-type (PB2-292I) and mutant (PB2-292V) H9N2 viruses were determined over 72 h in A549 and MDCK cells at an m.o.i. of 0.2 and 0.001, respectively. Wild-type PB2-292V virus was up to tenfold more productive in progeny virus than that of PB2-292I virus in A549 cells (*P*<0.05) (Fig. 5a). In MDCK cells, PB2-292V virus was also more productive by about tenfold relative to PB2-292I virus at 36, 48 and 60 h p.i. (*P*<0.01) (Fig. 5b). Thus, PB2-I292V H9N2 virus replicates to a higher titre than that of PB2-292V virus in mammalian cells.

### PB2-292V virus is more replication proficient and pathogenic than PB2-292I virus in mice

**Fig. 6.**
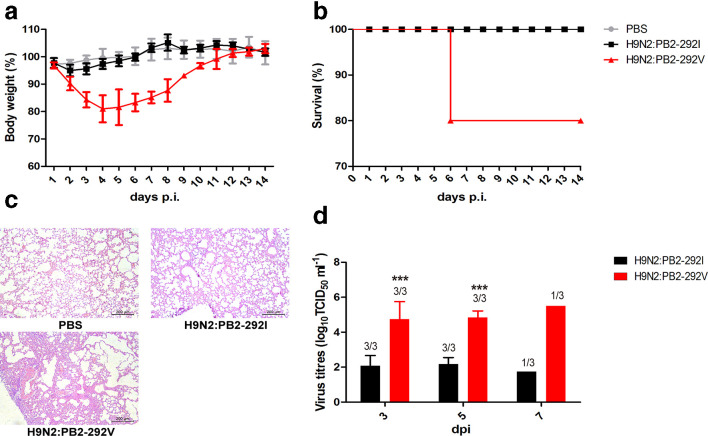
PB2-I292V mutation in H9N2 virus raised pathogenicity and viral replication in mice. Groups of BALB/c mice were inoculated with 106 TCID_50 _of the indicated in mice. Groups of BALB/c mice were inoculated with 10^6^ TCID_50_ of the indicated virus or mock infected with PBS. (a) Body weight changes of mice inoculated with H9N2:PB2-292I and H9N2:PB2-292V virus. Data are presented as means±sd of five individual mice. (b) Survival rates of mice. Mice infected with PB2-292V virus showed higher morbidity than PB2-292I virus. Mice with weight loss of more than 25 % were euthanized. (c) Hematoxylin and eosin (H&E) stained lung sections of mice infected with indicated virus at 5 days p.i. Scale bar, 200 μm. (d) Virus titres in lungs harvested from three mice per group at 3, 5 and 7 days p.i. Virus titres are means±sd of three individual mice. Statistical significance was based on two-way ANOVA (*P<0.05, **P<0.01, ***P<0.001).

Groups of mice were inoculated intranasally with PBS or 10^6^ TCID_50_ of each virus. During the 14 day period, H9N2:PB2-292I virus infected mice showed some weight loss of around 5.01 % at the start of infection with no mortality (Fig. 6a, b). H9N2:PB2-292V virus infection caused moderate weight loss (up to 20 %) and resulted in death in 1 out of 5 mice (Fig. 6a, b). H9N2:PB2-292I infected lungs showed mild changes while H9N2:PB2-292V infection resulted in severe pockets of interstitial pneumonia, characterized by alveolar interstitial consolidation and extensive inflammatory cell infiltration (Fig. 6c). Likewise, lung virus titres from PB2-292V virus infection were over 400-fold higher than those of PB2-292I virus at 3, 5 and 7 days p.i. (Fig. 6d). Collectively, PB2-292V in H9N2 virus improves virus replication and causes more severe infection in mice.

## Discussion

In this study, we identified a new mammalian adaptive mutation, PB2-I292V, which is highly prevalent in circulating H9N2, its reassortants and pdm H1N1 virus. PB2-292V mutation confers increased viral polymerase activity and enhanced inhibition of IFN-β induction in human cells. In the mouse model, PB2-292V mutation increases H9N2 virus replication with ensuing increase in disease severity. These findings provide an explanation for increased human infection cases of avian H9N2 virus and its reassortants.

Increased polymerase activity conferred by other mutations on PB2, such as PB2-E627K, has been shown to be crucial for AIVs to adapt to mammalian hosts [10]. PB2-627 residue is sited in several functional domains, including the NP protein-binding domain and the RNA-binding domain. PB2-E627K mutation alters polymerase activity by affecting the interaction of PB2 with NP [28] and exhibits higher binding affinity for RNA, in particular the 5′ vRNA promoter [29]. There are PB2 mutations not located in any known functional domain but exhibit significant mammalian adaptability, such as PA-356 residue [11]. In the present study, reside 292 in PB2 is not associated with any recognized functional domain, but is in proximity to the cap-binding domain [30]. Capped RNA recognition by the cap-binding domain of PB2 protein is important in the synthesis of viral mRNAs. PB2-I292V mutation could conceivably improve cap recognition and promote viral replication in human cells.

Interestingly, PB2-292I and PB2-292V mutations in H9N2 virus show no difference in viral polymerase activity in avian cells, suggesting that PB2-I292V mutation specifically promotes infectivity in mammalian cells. A similar situation is seen with the predominant PA-K356R and HA-Q226L mutations in H9N2 virus; they each specifically promote mammalian adaptation [11]. Other mutations, such as HA-A316S, however, were found to function efficiently in both avian and mammalian hosts [31].

Previous studies have demonstrated that growth and pathogenicity of influenza virus are influenced by not only viral polymerase activity but also host factors [27]. Type I IFN is known to be critical to the activation of host antiviral responses, and its inhibition leads to enhanced viral replication and virulence to host [32]. NS1 protein is the first well-characterized influenza viral protein shown to antagonize the host innate immune response and is a major virulence factor of multiple influenza virus strains [33, 34]. Additionally, PB1-F2 protein plays an important role in the increased virulence of H5N1 viruses through inhibiting the induction of type I IFN [35]. Notably, PB2 protein has been shown to inhibit IFN-β expression by interacting with MAVS [19]. Our results show that in human cells, H9N2 virus with PB2-292V mutation has the combined features of higher viral polymerase activity and stronger attenuation of host IFN-β response. Unlike the PB2-I292V mutation reported in the current study, the well-known human adaptive mutations of PB2-E627K and -D701N [10, 15] are rarely found in prevailing avian H9N2 viruses. Along with the propensity of PB2-292V mutation in H9N2 virus to reassort to other prevailing avian subtypes such as H7N9 and H10N8 viruses, their combined threat posed by its presence to human health could be significant. It is therefore necessary to closely monitor for the presence of PB2-292V, along with other known viral markers of virulence, to anticipate major outbreaks of severe disease in humans and other susceptible species.

## References

[1] Choi YK, Ozaki H, Webby RJ, Webster RG, Peiris JS (2004). Continuing evolution of H9N2 influenza viruses in southeastern China. J Virol.

[2] Sun Y, Liu J (2015). H9N2 influenza virus in China: a cause of concern. Protein Cell.

[3] Lin YP, Shaw M, Gregory V, Cameron K, Lim W (2000). Avian-to-human transmission of H9N2 subtype influenza A viruses: relationship between H9N2 and H5N1 human isolates. Proc Natl Acad Sci U S A.

[4] Xu C, Fan W, Wei R, Zhao H (2004). Isolation and identification of swine influenza recombinant A/Swine/Shandong/1/2003(H9N2) virus. Microbes Infect.

[5] Li X, Shi J, Guo J, Deng G, Zhang Q (2014). Genetics, receptor binding property, and transmissibility in mammals of naturally isolated H9N2 avian influenza viruses. PLoS Pathog.

[6] Lam TT-Y, Wang J, Shen Y, Zhou B, Duan L (2013). The genesis and source of the H7N9 influenza viruses causing human infections in China. Nature.

[7] Gao R, Cao B, Hu Y, Feng Z, Wang D (2013). Human infection with a novel avian-origin influenza A (H7N9) virus. N Engl J Med.

[8] Chen H, Yuan H, Gao R, Zhang J, Wang D (2014). Clinical and epidemiological characteristics of a fatal case of avian influenza A H10N8 virus infection: a descriptive study. The Lancet.

[9] Mehle A, Doudna JA (2009). Adaptive strategies of the influenza virus polymerase for replication in humans. Proc Natl Acad Sci U S A.

[10] Nilsson BE, te Velthuis AJW, Fodor E (2017). Role of the PB2 627 domain in influenza A virus polymerase function. J Virol.

[11] Xu G, Zhang X, Gao W, Wang C, Wang J (2016). Prevailing PA mutation K356R in avian influenza H9N2 virus increases mammalian replication and pathogenicity. J Virol.

[12] Labadie K, Dos Santos Afonso E, Rameix-Welti MA, van der Werf S, Naffakh N (2007). Host-range determinants on the PB2 protein of influenza A viruses control the interaction between the viral polymerase and nucleoprotein in human cells. Virology.

[13] Rameix-Welti MA, Tomoiu A, Dos Santos Afonso E, van der Werf S, Naffakh N (2009). Avian influenza A virus polymerase association with nucleoprotein, but not polymerase assembly, is impaired in human cells during the course of infection. J Virol.

[14] Luk GSM, Leung CYH, Sia SF, Choy KT, Zhou J (2015). Transmission of H7N9 influenza viruses with a polymorphism at PB2 residue 627 in chickens and ferrets. J Virol.

[15] Sediri H, Schwalm F, Gabriel G, Klenk HD (2015). Adaptive mutation PB2 D701N promotes nuclear import of influenza vRNPs in mammalian cells. Eur J Cell Biol.

[16] Bussey KA, Bousse TL, Desmet EA, Kim B, Takimoto T (2010). PB2 residue 271 plays a key role in enhanced polymerase activity of influenza A viruses in mammalian host cells. J Virol.

[17] Song W, Wang P, Mok BWY, Lau SY, Huang X (2014). The K526R substitution in viral protein pb2 enhances the effects of E627K on influenza virus replication. Nat Commun.

[18] Xiao C, Ma W, Sun N, Huang L, Li Y (2016). PB2-588 V promotes the mammalian adaptation of H10N8, H7N9 and H9N2 avian influenza viruses. Sci Rep.

[19] Graef KM, Vreede FT, Lau YF, McCall AW, Carr SM (2010). The PB2 subunit of the influenza virus RNA polymerase affects virulence by interacting with the mitochondrial antiviral signaling protein and inhibiting expression of beta interferon. J Virol.

[20] Tang J, Qu LK, Zhang J, Wang W, Michaelson JS (2006). Critical role for Daxx in regulating MDM2. Nat Cell Biol.

[21] Sun Y, Qin K, Wang J, Pu J, Tang Q (2011). High genetic compatibility and increased pathogenicity of reassortants derived from avian H9N2 and pandemic H1N1/2009 influenza viruses. Proc Natl Acad Sci U S A.

[22] Reed LJ, Muench H (1938). A simple method of estimating fifty per cent endpoints. Am J Epidemiol.

[23] Zhang Y, Sun Y, Sun H, Pu J, Bi Y (2012). A single amino acid at the hemagglutinin cleavage site contributes to the pathogenicity and neurovirulence of H5N1 influenza virus in mice. J Virol.

[24] Czudai-Matwich V, Otte A, Matrosovich M, Gabriel G, Klenk HD (2014). PB2 mutations D701N and S714R promote adaptation of an influenza H5N1 virus to a mammalian host. J Virol.

[25] Hu M, Yuan S, Zhang K, Singh K, Ma Q (2017). PB2 substitutions V598T/I increase the virulence of H7N9 influenza A virus in mammals. Virology.

[26] Naffakh N, Tomoiu A, Rameix-Welti MA, van der Werf S (2008). Host restriction of avian influenza viruses at the level of the ribonucleoproteins. Annu Rev Microbiol.

[27] Zhao Z, Yi C, Zhao L, Wang S, Zhou L (2014). PB2-588I enhances 2009 H1N1 pandemic influenza virus virulence by increasing viral replication and exacerbating PB2 inhibition of beta interferon expression. J Virol.

[28] Rameix-Welti MA, Tomoiu A, Dos Santos Afonso E, van der Werf S, Naffakh N (2009). Avian influenza A virus polymerase association with nucleoprotein, but not polymerase assembly, is impaired in human cells during the course of infection. J Virol.

[29] Kuzuhara T, Kise D, Yoshida H, Horita T, Murazaki Y (2009). Structural basis of the influenza A virus RNA polymerase PB2 RNA-binding domain containing the pathogenicity-determinant lysine 627 residue. J Biol Chem.

[30] Mänz B, Schwemmle M, Brunotte L, Benjamin MN, Martin S (2013). Adaptation of avian influenza A virus polymerase in mammals to overcome the host species barrier. J Virol.

[31] Sun Y, Tan Y, Wei K, Sun H, Shi Y (2013). Amino acid 316 of hemagglutinin and the neuraminidase stalk length influence virulence of H9N2 influenza virus in chickens and mice. J Virol.

[32] García-Sastre A, Biron CA (2006). Type 1 interferons and the virus-host relationship: a lesson in détente. Science.

[33] Meunier I, von Messling V, Von MV (2011). NS1-mediated delay of type I interferon induction contributes to influenza a virulence in ferrets. J Gen Virol.

[34] Forbes NE, Ping J, Dankar SK, Jia JJ, Selman M (2012). Multifunctional adaptive NS1 mutations are selected upon human influenza virus evolution in the mouse. PLoS One.

[35] Leymarie O, Jouvion G, Hervé PL, Chevalier C, Lorin V (2013). Kinetic characterization of PB1-F2-mediated immunopathology during highly pathogenic avian H5N1 influenza virus infection. PLoS One.

